# Optic Nerve Head Cotton Wool Spots Mimicking Inflammatory Optic Neuropathy in Central Retinal Vein Occlusion

**DOI:** 10.7759/cureus.114074

**Published:** 2026-08-06

**Authors:** Carolina A Jimenez Soto, Ninel L Gregori, Byron L Lam, Mariam Vila-Delgado, Luma Al-Attar

**Affiliations:** 1 School of Medicine, University of Puerto Rico, San Juan, USA; 2 Ophthalmology, University of Miami, Miami, USA; 3 Ophthalmology, University of Puerto Rico, San Juan, USA

**Keywords:** central retinal vein occlusion (crvo), cotton wool spot, oct (optical coherence tomography), optic nerve, optic nerve infiltration

## Abstract

Central retinal vein occlusion (CRVO) typically presents with optic disc edema, venous dilation, and widespread retinal hemorrhages; however, atypical optic nerve head findings can mimic neuro-inflammatory or infiltrative disease, posing a diagnostic challenge.

We report a 67-year-old woman with diabetes mellitus, rheumatoid arthritis, hypertension, and hyperlipidemia who presented for routine evaluation without visual symptoms and was found to have non-proliferative diabetic retinopathy bilaterally and clinical signs of non-ischemic CRVO in the left eye, including optic disc edema, hyperemia, and intraretinal hemorrhages. Optical coherence tomography (OCT) demonstrated mild macular thickening, and fluorescein angiography (FA) showed scattered hyperfluorescent foci with minimal leakage. Two months after intravitreal faricimab injection, macular edema resolved and visual acuity improved; however, as disc edema subsided, new large whitish nodular lesions emerged on the optic nerve head inferiorly, nasally, and superiorly, corresponding to hyperreflective deposits on OCT. Given the atypical appearance, brain and orbits MRI with contrast, lumbar puncture, and infectious and inflammatory laboratory testing were performed, all of which were unremarkable, with no evidence of infiltrative, demyelinating, or vasculitic disease. Repeat FA revealed no vessel wall staining or leakage to suggest retinal vasculitis. The nodular optic nerve lesions were ultimately determined to be prominent cotton wool spots (CWS) associated with CRVO, presenting in an unusual peripapillary distribution that spared the maculopapillary bundle, consistent with localized inner retinal ischemia and disruption of axoplasmic transport. The lesions resolved over several months without development of optic atrophy or collateral vessels, and final visual acuity returned to 20/20 with normal visual fields.

This case highlights that large CWS on the optic nerve head in non-ischemic CRVO can closely mimic neuro-inflammatory or infiltrative processes and that multimodal imaging combined with multidisciplinary evaluation is essential to avoid misdiagnosis and unnecessary treatment.

## Introduction

Central retinal vein occlusion (CRVO) is the second most common retinal vascular disorder after diabetic retinopathy and remains a significant cause of visual morbidity. It arises from thrombotic obstruction of the central retinal vein, typically at or just posterior to the lamina cribrosa, where the vessel lumen narrows and blood flow accelerates, promoting turbulence and endothelial injury [1]. The resulting rise in venous pressure produces venous congestion, intraretinal hemorrhage, capillary nonperfusion, and macular edema. Common risk factors include advanced age, hypertension, diabetes mellitus, hyperlipidemia, glaucoma, and prothrombotic states such as hyperhomocysteinemia or antiphospholipid antibodies [1].

CRVO is conventionally divided into non-ischemic and ischemic subtypes, a distinction based on functional and morphologic criteria (visual acuity, relative afferent pupillary defect, visual field, electroretinography, and the extent of retinal nonperfusion on fluorescein angiography (FA)) [2]. Non-ischemic CRVO, which accounts for roughly 80% of acute presentations, is characterized by better presenting acuity, little or no capillary nonperfusion, and macular edema from perifoveal capillary leakage; visual acuity is 20/100 or better in approximately 78% of eyes at presentation and in 83% after resolution of macular edema [3,4]. Ischemic CRVO, defined in the Central Vein Occlusion Study as 10 or more disc areas of retinal nonperfusion, presents with poor acuity, a relative afferent pupillary defect, extensive confluent hemorrhages, and a high risk of iris neovascularization and neovascular glaucoma; only about 1% of such eyes present with acuity of 20/100 or better and 12% achieve this level at the final follow-up [3,4]. This distinction is not static: up to one-third of non-ischemic eyes convert to the ischemic subtype within three years, most often in the first four months, so serial reassessment is essential [3,5].

The classic fundus appearance of CRVO comprises hemorrhages in all four quadrants, dilated and tortuous retinal veins, optic disc edema, and cotton wool spots (CWS) [1]. CWS are not exudates but focal infarcts of the retinal nerve fiber layer: interruption of the retinal arteriolar supply halts axoplasmic transport, and the accumulation of degenerating axoplasmic organelles forms cytoid bodies that appear ophthalmoscopically as fluffy, white, feathery-edged superficial lesions with indistinct margins [6]. They are typically small, few in number, distributed along the arcades in the posterior pole, and fade over weeks as the acute process resolves [7]. Their presence, particularly when abundant and accompanied by large confluent hemorrhages, is a recognized marker of retinal ischemia [2,7]. Notably, a transient increase in CWS has been described following intravitreal anti-VEGF therapy for CRVO-related macular edema, generally without adverse effect on visual recovery [6]. When CWS instead appear as large, nodular lesions concentrated in the peripapillary region or overlying the optic nerve head itself, the fundus appearance can be difficult to distinguish from primary disease of the optic nerve. The relevant differential includes inflammatory and demyelinating optic neuritis, infectious neuroretinitis, granulomatous or infiltrative optic neuropathy (sarcoidosis, lymphoma, leukemic or metastatic infiltration), non-arteritic anterior ischemic optic neuropathy, and papilledema, entities in which peripapillary opacification, fluid, and disc elevation overlap substantially with the resolving disc changes of CRVO and are a recognized source of misdiagnosis and inappropriate management [8]. The overlap is compounded when disc swelling is prominent, as in the papillophlebitis pattern of CRVO described in younger patients, and in patients with autoimmune or inflammatory comorbidity, in whom pretest suspicion for a neuro-inflammatory cause is heightened and extensive, costly systemic and neuroimaging evaluation may be triggered. We report a case of non-ischemic CRVO in a 67-year-old patient with multiple vascular and autoimmune risk factors who developed large nodular CWS overlying the optic nerve head during resolution of disc edema, simulating a primary optic nerve or infiltrative process. This case illustrates the importance of recognizing this atypical morphology of an otherwise common finding, so that the diagnosis can be secured on the basis of the accompanying retinal vascular signs and multimodal imaging, avoiding unnecessary investigation and directing management toward the macular edema and systemic vascular risk factors that determine outcome.

This article was previously presented as a meeting abstract at the 2026 EnVision Summit on February 13-16, 2026 and 46th Annual Research and Education Forum of the Medical Sciences Campus of the University of Puerto Rico on March 25-26, 2026.

## Case presentation

A 67-year-old woman was initially seen by her general ophthalmologist for routine eye screening with no visual symptoms. Her medical history includes diabetes mellitus (HbA1c 6.5%), rheumatoid arthritis, hypertension, and hyperlipidemia. Current medications include methotrexate, losartan-hydrochlorothiazide, metoprolol, simvastatin, and insulin therapy.

**Figure 1:**
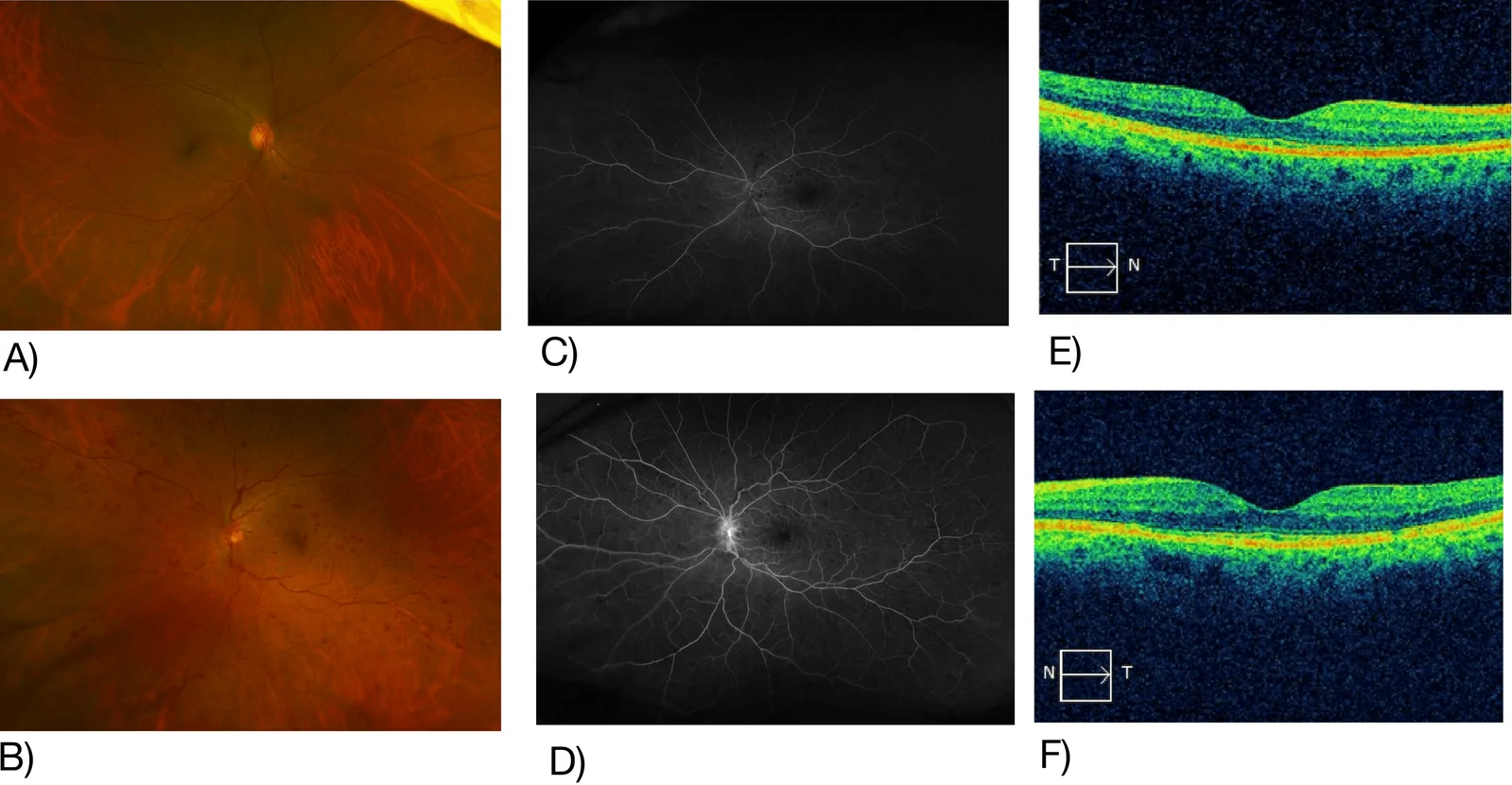
Multimodal imaging at initial presentation. Multimodal imaging at presentation (Day 1). (A) Color fundus photograph of the right eye, unremarkable. (B) Color fundus photograph of the left eye demonstrating optic disc edema with peripapillary and scattered intraretinal hemorrhages and venous tortuosity. (C) Early-phase fluorescein angiography of the left eye (35 seconds) demonstrating scattered pinpoint hyperfluorescence without significant capillary nonperfusion. (D) Late-phase fluorescein angiography of the left eye (1 minute 52 seconds) showing mild late leakage at the optic disc with blocked fluorescence from overlying hemorrhages. (E) OCT of the right eye showing normal foveal contour without intraretinal or subretinal fluid. (F) OCT of the left eye demonstrating mild diffuse noncystic retinal thickening without significant macular edema.

Dilated fundus examination with color fundus photography at presentation demonstrated a normal right eye (Figure 1A). The left eye showed optic disc edema with peripapillary and scattered intraretinal hemorrhages extending into the posterior pole and mid-periphery, with venous tortuosity (Figure 1B). FA of the left eye demonstrated early scattered pinpoint areas of hyperfluorescence without significant capillary nonperfusion (Figure 1C, 35 seconds), with late mild leakage at the optic disc and blocked fluorescence corresponding to the overlying retinal hemorrhages (Figure 1D, 1 minute 52 seconds). Optical coherence tomography (OCT) of the right eye demonstrated a normal foveal contour without intraretinal or subretinal fluid (Figure 1E). OCT of the left eye showed mild diffuse noncystic retinal thickening relative to the right eye, without significant macular edema (Figure 1F).

The patient was diagnosed with a non-ischemic CRVO in the left eye and severe non-proliferative diabetic retinopathy in both eyes.

**Figure 2:**
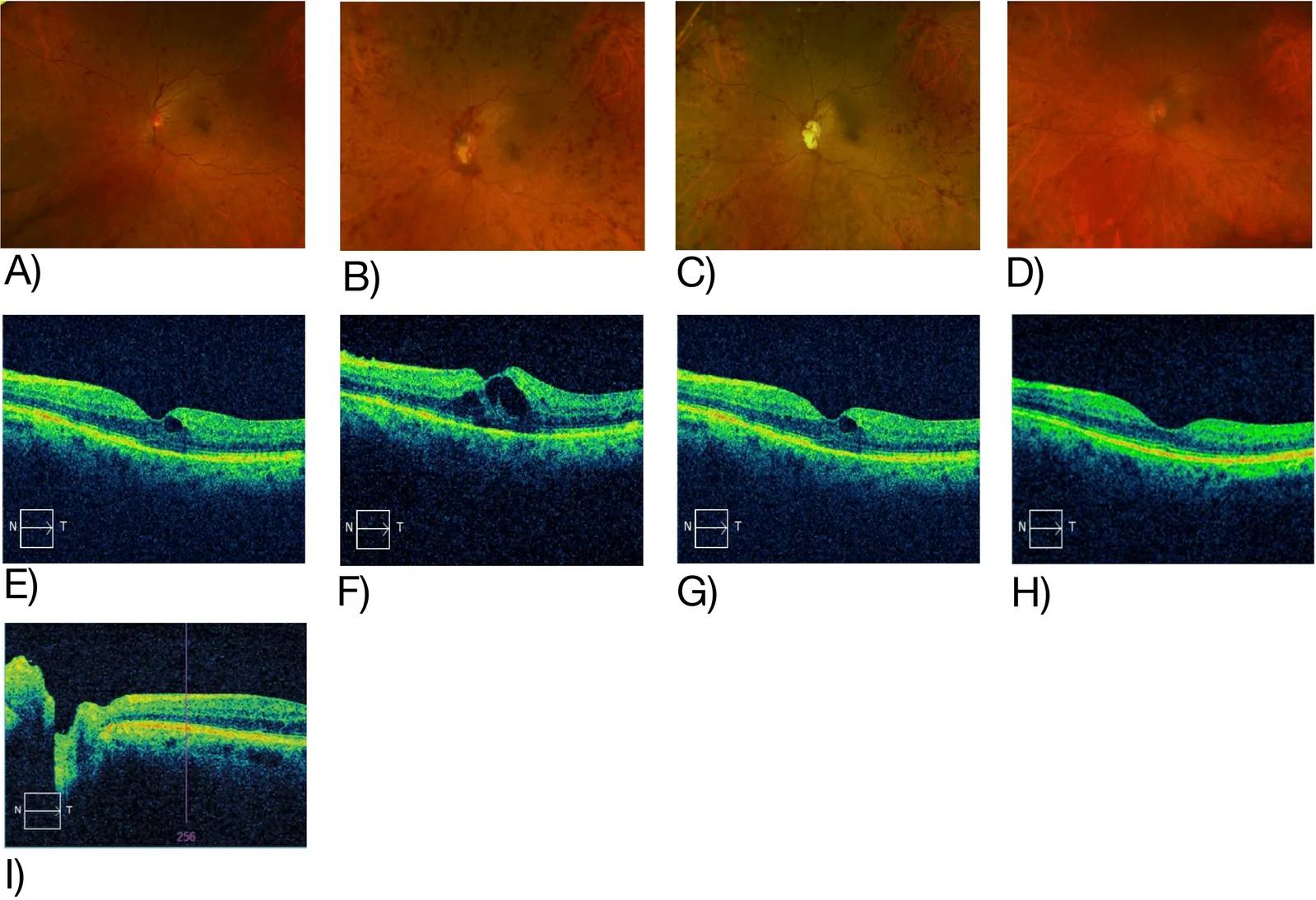
Longitudinal progression of fundus photographs and OCT findings. Longitudinal color fundus photography and OCT findings. (A–D) Serial color fundus photographs of the left eye obtained at presentation (Day 1), Month 1, Month 3, and Month 5, respectively, demonstrating gradual resolution of optic disc edema and retinal hemorrhages with increasing prominence and subsequent resolution of peripapillary cotton wool spots. (E–H) Serial macular OCT images of the right eye obtained at Month 1, Month 2, Month 3, and Month 5, showing interval development and subsequent resolution of macular edema. (I) OCT of the right optic nerve obtained at Month 5 demonstrating hyperreflective peripapillary lesions corresponding to cotton wool spots.

Serial color fundus photography of the left eye documented the clinical course over five months (Figures 2A-2D). At presentation, there was optic disc edema with peripapillary and scattered intraretinal hemorrhages (Figure 2A). At Month 1, peripapillary CWS had become more prominent, with persistent scattered intraretinal hemorrhages and residual disc edema (Figure 2B). At Month 3, confluent peripapillary CWS remained evident overlying the disc margin, with gradual resolution of the retinal hemorrhages (Figure 2C). By Month 5, the disc edema, CWS, and hemorrhages had largely resolved (Figure 2D).

Serial macular OCT over the same interval demonstrated the interval development and subsequent resolution of macular edema (Figures 2E-2H). At Month 1, the foveal contour was preserved with only minimal intraretinal fluid (Figure 2E). At Month 2, there was interval development of cystoid intraretinal fluid with associated foveal thickening (Figure 2F). By Month 3, the intraretinal cystoid spaces had substantially decreased (Figure 2G), and by Month 5, the macula was anatomically dry with a restored foveal contour and preserved outer retinal architecture (Figure 2H). OCT of the optic nerve obtained at Month 5 demonstrated resolution of the optic disc elevation with residual hyperreflective peripapillary lesions corresponding to CWS (Figure 2I).

**Figure 3:**
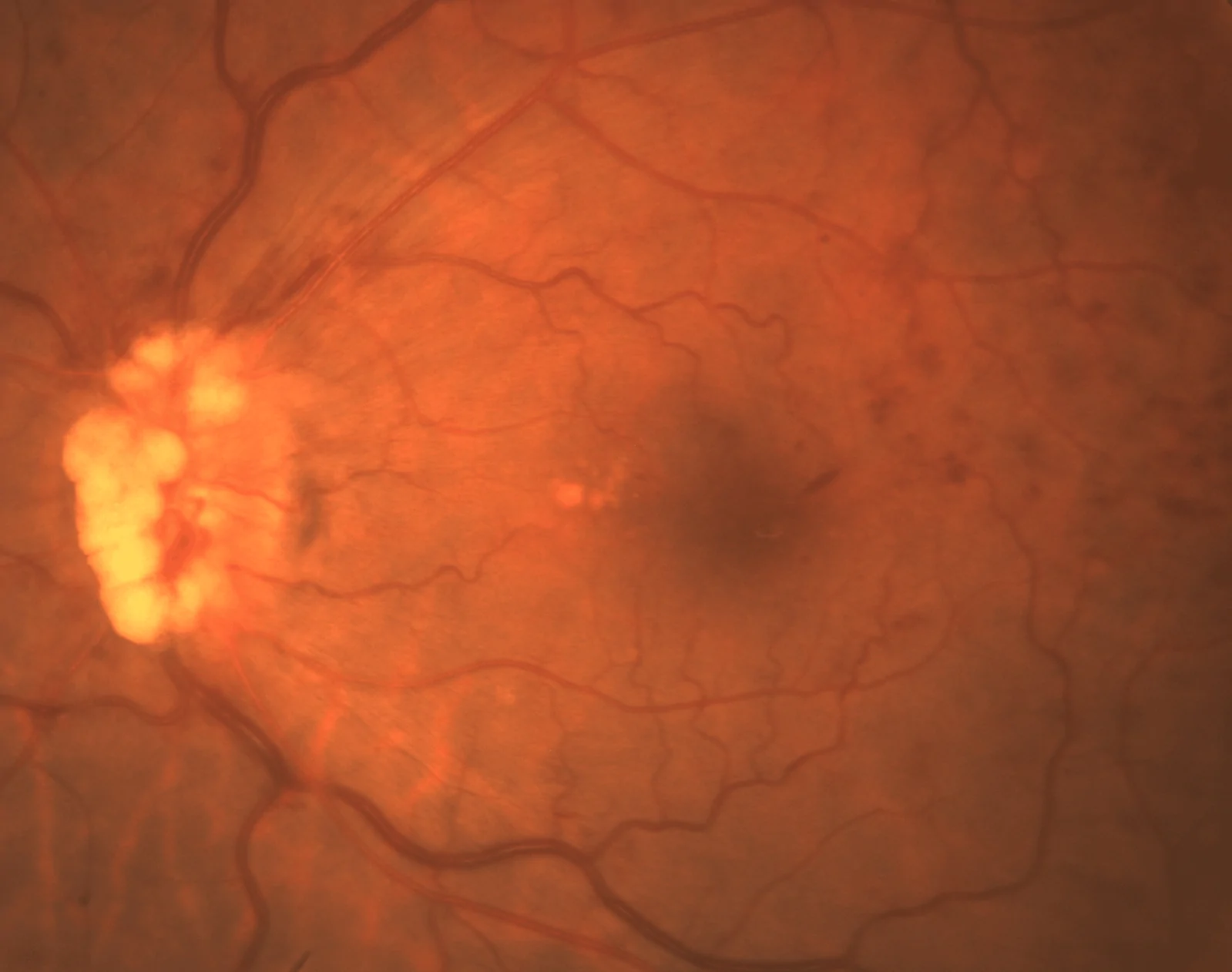
Color fundus photographs obtained using Topcon imaging.

**Figure 4:**
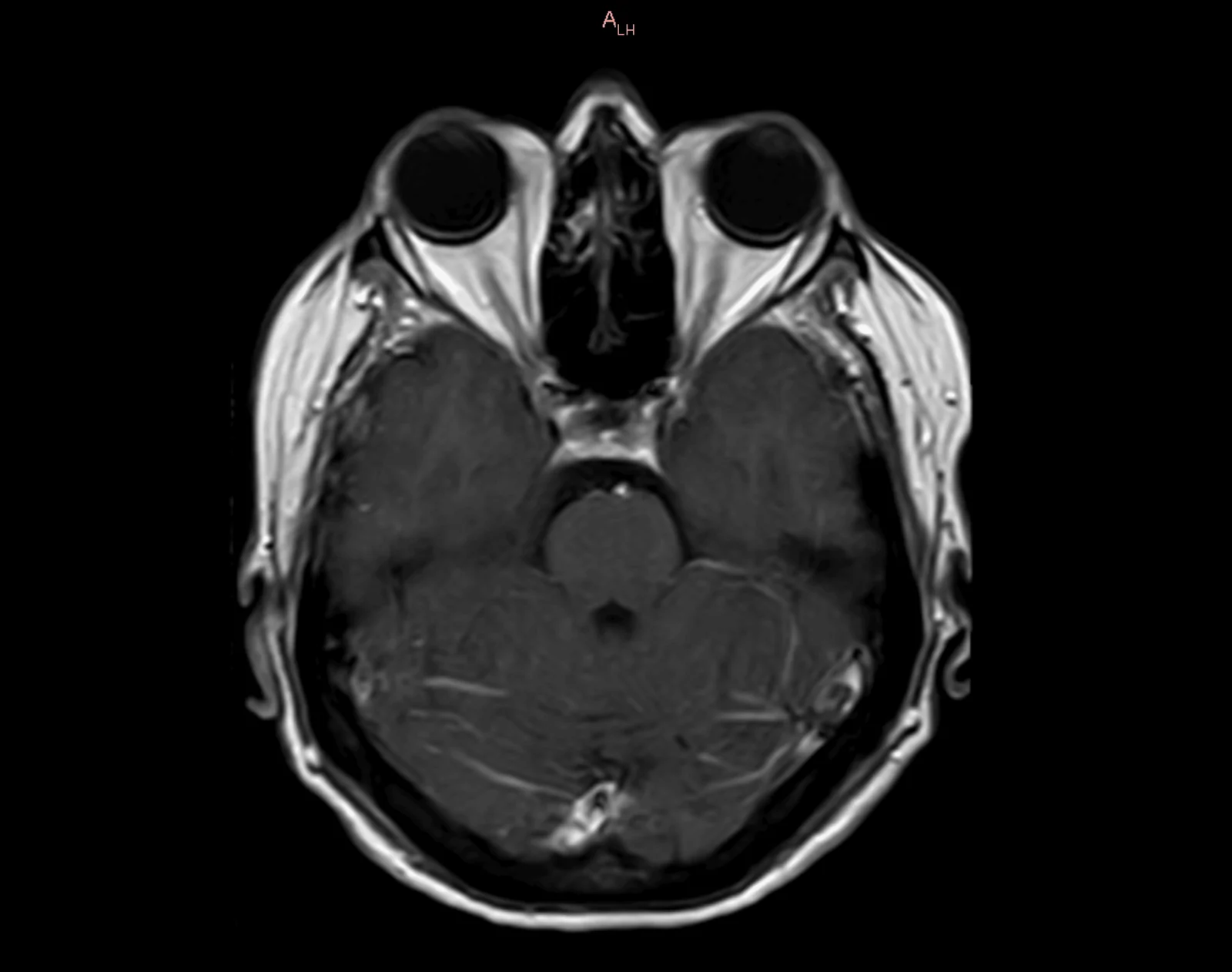
Axial post-contrast T1-weighted MRI of the brain at the level of the posterior fossa and orbits. The bilateral globes and retro-orbital structures appear intact without evidence of infiltrative disease.

Given the atypical optic nerve findings (Figure 3), a neuro-ophthalmologic evaluation was begun to rule out a primary optic nerve process. This included inflammatory, infiltrative, infectious, or demyelinating etiologies. Brain and orbit MRI (Figure 4) with gadolinium contrast demonstrated no optic nerve enhancement, no orbital mass lesion, and no intracranial abnormality. Lumbar puncture revealed normal opening pressure, cell counts, protein, glucose, and cytology. Laboratory studies included a complete blood count with automated differential, comprehensive metabolic panel, angiotensin-converting enzyme (ACE) level, and rapid plasma reagin (RPR). All results were within normal limits; specifically, the ACE level was not elevated, and RPR was nonreactive. Humphrey visual field testing was full in the left eye (OS), without any visual field defects.

The neuro-ophthalmic evaluation was unremarkable, with no evidence of infiltrative, demyelinating, or vasculitic disease. The nodular optic nerve lesions were determined to be prominent CWS, presenting in an unusual peripapillary distribution that spared the maculopapillary bundle. This distribution was consistent with localized retinal ischemia and disruption of axoplasmic transport resulting in debris accumulation within the nerve fiber layer. Final acuity returned to 20/20, and visual fields returned to normal. 

## Discussion

This case illustrates an atypical manifestation of non-ischemic CRVO in which prominent peripapillary CWS on the optic nerve head mimicked an inflammatory or infiltrative optic neuropathy, prompting an extensive neuro-ophthalmic workup. While CWS are a well-recognized feature of CRVO, their occurrence as large, nodular, optic nerve-predominant lesions during the resolution phase of disc edema is unusual and has not been well described in the literature.

CWS in CRVO are typically small, scattered throughout the posterior pole and clinically inconspicuous [4]. Hayreh and Zimmerman, in a comprehensive study with 562 CRVO patients, demonstrated that retinal hemorrhages, optic nerve edema, and CWS were initially more marked in ischemic CRVO than in non-ischemic CRVO, and that these findings took longer to resolve in the ischemic subtype [5]. In this case, the patient had non-ischemic CRVO with preserved visual acuity, no relative afferent pupillary defect, and minimal retinal non-perfusion on FA. However, the CWS in this patient were unusually large and concentrated on the optic nerve head rather than scattered across the posterior pole, creating a clinical appearance that diverged from the typical CRVO presentation described in major reviews [1].

The pathophysiology of CWS has been extensively studied. McLeod et al. demonstrated in a landmark experimental study that CWS represent accumulations of cytoplasmic debris within the retinal nerve fiber layer caused by obstruction of both anterograde and retrograde axoplasmic transport in ganglion cell axons, rather than true retinal nerve fiber layer infarcts [4]. In a subsequent analysis, McLeod proposed that CWS should be regarded as "sentinels" of retinal nerve fiber layer pathology, forming at the boundaries of ischemic zones rather than within the ischemic territory itself [6]. In pre-proliferative diabetic retinopathy, for example, CWS characteristically form a C-shaped chain nasal to the disc and around the macula, serving as sentinels of ischemia affecting the retinal mid-periphery. The distribution pattern observed in the present case, with CWS concentrated inferiorly, nasally, and superiorly on the optic nerve head while sparing the maculopapillary bundle, is consistent with this sentinel concept, suggesting that the CWS formed at the boundaries of localized inner retinal ischemia surrounding the optic disc in the setting of venous congestion. 

The OCT findings in this case are consistent with prior studies characterizing the imaging features of CWS. Kozak et al. dated that acute CWS show a hyperreflective pattern on OCT corresponding to the accumulation of axoplasmic debris, and that this hyperreflectivity persists even after the CWS become ophthalmoscopically invisible, a finding termed the "hyperreflective sign" [7]. Coady et al. further characterized the spectral-domain OCT appearance of acute ischemic retinal whitening in various retinal vascular occlusions, including CRVO, demonstrating inner retinal hyperreflectivity and thickening as hallmarks of ischemic injury [8]. In the present case, the hyperreflective areas on OCT corresponding to the optic nerve head lesions were consistent with these described OCT features of CWS, helping to confirm their identity as ischemic rather than inflammatory or infiltrative lesions.

However, the differential diagnosis of optic nerve head lesions in this clinical context is broad. Biousse and Newman outlined the major categories of optic neuropathy, including inflammatory (optic neuritis, neuromyelitis optica, systemic autoimmune diseases), infectious (syphilis, tuberculosis), compressive/infiltrative (neoplastic, leptomeningeal), ischemic (arteritic and non-arteritic anterior ischemic optic neuropathy), and toxic/nutritional etiologies [9]. In the present case, the patient's concurrent rheumatoid arthritis and immunosuppressive therapy with methotrexate heightened clinical suspicion for an autoimmune or infectious optic neuropathy, necessitating a comprehensive workup.

Retinal vasculitis associated with systemic autoimmune diseases, particularly systemic lupus erythematosus, can produce peripapillary CWS and retinal hemorrhages that may closely resemble CRVO [10]. McCluskey and Powell described retinal vasculitis in SLE as an arteriolar vasculitis that produces multiple retinal CWS and occasionally larger retinal arterial occlusions, emphasizing the need to distinguish vasculitis from hypertensive vascular changes [10]. In the present case, FA showed no vessel wall staining or leakage, findings that would be expected in retinal vasculitis [10]. This absence of angiographic vasculitis, combined with negative serologic testing, normal MRI, and unremarkable cerebrospinal fluid analysis, effectively excluded inflammatory, infectious, and infiltrative etiologies.

The favorable outcome in this case is consistent with the natural history of non-ischemic CRVO. Scott et al. noted that eyes with non-ischemic CRVO presenting with good vision tend to retain good vision, in contrast to ischemic CRVO where progressive retinal non-perfusion and neovascular complications are common [1]. The CWS in the present case resolved over several months without development of optic atrophy, consistent with the known natural history of CWS resolution in retinal vascular disease, which typically occurs within weeks to months in non-diabetic patients but may persist for one to two years in diabetic patients. Importantly, no optic disc collateral vessels developed in this patient. Arrigo et al. demonstrated that collateral vessel development in CRVO is associated with worse visual and anatomic outcomes, and their absence in this case is consistent with the favorable prognosis of non-ischemic CRVO [11]. 

The role of multimodal imaging in this case deserves emphasis. Biousse et al. highlighted that multimodality optic nerve imaging is especially useful when evaluating patients with possible optic disc edema, as OCT with enhanced depth imaging complements fundus photography by providing direct imaging of deeper optic nerve structures [12]. In the present case, the combination of serial fundus photography, OCT, and FA was essential for characterizing the temporal evolution of the optic nerve findings, confirming the ischemic nature of the lesions, and excluding vasculitis. This multimodal approach, combined with neuroimaging and laboratory evaluation, allowed for confident attribution of the optic nerve findings to CRVO-associated CWS rather than a primary optic nerve process.

This case has several clinical implications. First, clinicians should be aware that CWS in CRVO can occasionally present as large, nodular, optic nerve-predominant lesions during the resolution phase of disc edema, creating a clinical appearance that may be mistaken for inflammatory or infiltrative optic neuropathy. Second, in patients with concurrent autoimmune disease, a systematic approach using multimodal imaging and targeted laboratory evaluation can efficiently distinguish CRVO-associated CWS from conditions requiring immunosuppressive or other disease-specific therapy. Finally, the favorable visual outcome in this case, with complete CWS resolution, no optic atrophy, and preserved visual acuity and visual fields, underscores the benign nature of this finding when correctly identified and supports a conservative management approach with close monitoring.

## Conclusions

This case demonstrates an unusual presentation of non-ischemic CRVO in which large peripapillary CWS developed during the resolution of optic disc edema and mimicked a neuro-inflammatory or infiltrative optic neuropathy. The atypical distribution, concentrated inferiorly, nasally, and superiorly on the optic nerve head while sparing the maculopapillary bundle, is consistent with the sentinel concept of CWS formation at the boundaries of localized inner retinal ischemia. The extensive diagnostic evaluation, including neuroimaging, cerebrospinal fluid analysis, infectious serologies, and rheumatologic assessment, was unrevealing, and multimodal imaging with OCT and FA ultimately confirmed the ischemic nature of the lesions and excluded retinal vasculitis.

Clinicians should be aware that as disc edema resolves in CRVO, previously obscured CWS may become clinically apparent, creating the impression of new optic nerve pathology. In patients with concurrent systemic inflammatory disease or immunosuppressive therapy, this presentation may heighten suspicion for alternative etiologies. However, peripapillary CWS should remain in the differential diagnosis of atypical optic nerve findings in CRVO, and a systematic multimodal imaging approach can efficiently distinguish these benign ischemic lesions from conditions requiring disease-specific therapy. The favorable outcome in this case, with complete CWS resolution, no optic atrophy, and preserved visual acuity and visual fields, underscores the benign prognosis of this finding when correctly identified and supports close monitoring rather than aggressive intervention.
